# Influence of various disinfection methods on the flexural strength of additively manufactured denture base resins: an in vitro study

**DOI:** 10.1186/s12903-026-08749-x

**Published:** 2026-06-10

**Authors:** Faris A. Alshahrani, Zainab Albazroun, Ferdoos Alhalimi, Fatimah A. Aldobais, Qutuf Alzaid, Murtadha Albin Hamdhah, Soban Q Khan, Sultan Akhtar, Hamad S AlRumaih, Mohammed M. Gad

**Affiliations:** 1 Department of Substitutive Dental Sciences, College of Dentistry, Imam Abdulrahman Bin Faisal University, P.O. Box 1982, Dammam, 31441 Saudi Arabia; 2 College of Dentistry, Imam Abdulrahman Bin Faisal University, P.O. Box 1982, Dammam, 31441 Saudi Arabia; 3 Department of Dental Education, College of Dentistry, Imam Abdulrahman Bin Faisal University, P.O. Box 1982, Dammam, 31441 Saudi Arabia; 4 Department of Biophysics, Institute for Research and Medical Consultations, Imam Abdulrahman Bin Faisal University, P.O. Box 1982, Dammam, 31441 Saudi Arabia

**Keywords:** 3D printing, Removable dental prosthesis, Disinfection, Mechanical testing

## Abstract

**Background:**

With the advancement of technology for digitally fabricated removable dental prostheses, protocols for the disinfection of three-dimensional (3D)-printed dentures are lacking. Therefore, this study aimed to assess and compare the effects of different disinfection protocols on the flexural strength (FS) of 3D-printed denture base resins.

**Materials and methods:**

A total of 200 bar-shaped samples (64 × 10 × 3.3 mm) were fabricated via two types of 3D-printed denture base resins: DentaBase (DB) and Denture 3D+ (D). According to the disinfectant method, each resin sample was divided into five groups (*n* = 20). The samples were then further subdivided based on the duration of disinfection, either 90 days or 180 days. One control group was kept in water, while the other four groups were disinfected using a effervescent denture cleanser tablets (EDC), microwave (MW), ultraviolet light (UV), or ultrasonic (US). The flexural strength (FS) was measured. The collected data were statistically analyzed via three-way ANOVA, and post hoc Tukey’s test was used for data analysis (α = 0.05).

**Results:**

For Denture 3D+, FT significantly reduced FS at 90- and 180-day intervals (*p* < 0.05). UV disinfection maintained the highest FS at 90 days (72.1 MPa, *p* < 0.013); however, both the US and MW methods resulted in significant reductions by 180 days (*p* < 0.05). In the DentaBase, FT and MW disinfection caused significant FS reductions at 90 days (*p* < 0.001). By 180 days, FT, US, and MW disinfection methods led to significant decreases in FS (*p* < 0.001). However, UV light did not have a significant effect on FS at either point (*p* = 0.22).

**Conclusion:**

UV disinfection proved to be the most suitable method for maintaining the mechanical integrity of 3D-printed denture base resins, while chemical, microwave, and ultrasonic methods should be used with caution due to their deteriorating effects on flexural strength.

## Introduction

Complete dentures have long been the gold standard for treating edentulous people [1]. Recently, advances in both technology and science have made digitization possible through the use of subtractive manufacturing (SM, milled) or additive manufacturing (AM, 3D-printed) methods, which offer several potential benefits, including faster denture production, fewer steps in the work process, reduced costs, and possible errors [2]. The maintenance of removable dental prostheses is crucial, especially for denture wearers. Proper maintenance resulted in improved oral health, and the prosthesis exhibited clinically acceptable esthetics and was odor free. For proper oral health and good oral hygiene among denture wearers, establishing an effective disinfection protocol that is clinically viable, cost-effective, and straightforward to use is necessary [3].

A disinfection technique should ideally function properly without impacting the characteristics of the denture base and teeth [4]. However, disinfection has been reported to cause changes in specific material characteristics [4, 5]. For example, the flexural strength is negatively affected when alcohol-based disinfectants are used [5]. In addition, surface property changes, such as staining or bleaching, result from the long-term use of chlorhexidine and sodium hypochlorite disinfectants, respectively [6]. Therefore, alternative disinfectant methods with effective disinfection methods that do not deteriorate resin properties are recommended [7].

Microwaves are recommended as fast, effective, chemical-free, and low-cost alternatives to chemical disinfection for addressing the issues that arise [7]. Microwaves depend mainly on irradiating with electromagnetic energy at different wavelengths [5]. Although the effectiveness of disinfection via microwave irradiation has not been well reported [8], the precise mechanism of disinfection remains unclear. However, two claims have been reported: thermal or nonthermal character effects could be significant [5].

Ultrasonic waves are frequently used in dentistry for instrument cleaning and scaling [9]. Numerous gas bubbles are created when high-frequency sound waves from an ultrasonic cleaner generator pass through an ultrasonic cleaning solution [10]. When these gas bubbles burst, cavitation occurs, producing a large amount of impact energy, and a high-energy liquid stream collides with the surface of the object being cleaned [7]. Some studies have shown that the use of an ultrasonic cleaner significantly affects the disinfection of acrylic resin [11, 12]. On the other hand, Ultraviolet (UV) light is now being explored as a promising approach for treating infections caused by biofilms [13]. UV radiation is categorized based on wavelength into three types: UV-A (315–400 nm), UV-B (280–315 nm), and UV-C (100–280 nm) [14]. Numerous studies have demonstrated the effectiveness of various UV-C light exposure intervals and doses in reducing the adhesion and viability of *C. albicans* [8, 15]. Additionally, extended UV-A exposure durations can result in significant bactericidal or bacteriostatic effects, even when applied from relatively long distances [16]. Flexural strength refers to the stress a material could endure just before it breaks. It is a critical mechanical property used to evaluate denture base materials subjected to repeated masticatory stresses that may lead to deformation or fracture over time [17, 18]. The denture base must have specific qualities to accommodate occlusal forces since the greater the value is, the stiffer the material [19]. To ensure clinical performance and durability, denture base materials must comply with the minimum requirements set by International Organization for Standardization (ISO) 20795-1:2013, which specify a flexural strength of at least 65 MPa [17].

Recently, three-dimensional (3D) printed denture base materials have been introduced to the market; however, the effects of different disinfection methods on these materials remain largely unexplored, and no standardized disinfection protocols have been established for 3D-printed resins [20]. This study is original in its long-term evaluation of multiple disinfection techniques on two distinct 3D-printed denture base resins, providing new insights into how various disinfection approaches influence their flexural strength and durability. The findings contribute to establishing evidence-based guidelines for the maintenance and clinical longevity of 3D-printed denture bases. The null hypotheses of this study were as follows: H₀₁: The type of material has no significant effect on the flexural strength of 3D-printed denture base resins. H₀₂: The disinfection method has no significant effect on the flexural strength of 3D-printed denture base resins. H₀₃: The time interval has no significant effect on the flexural strength of 3D-printed denture base resins. H₀₄: There is no significant interaction effect among the type of material, disinfection method, and time on the flexural strength of 3D-printed denture base resins.

## Materials and methods

This study was approved by deanship of Scientific Research and Innovation Imam Abdulrahman Bin Faisal University (Approval date: 7/5/2024).

**Fig. 1:**
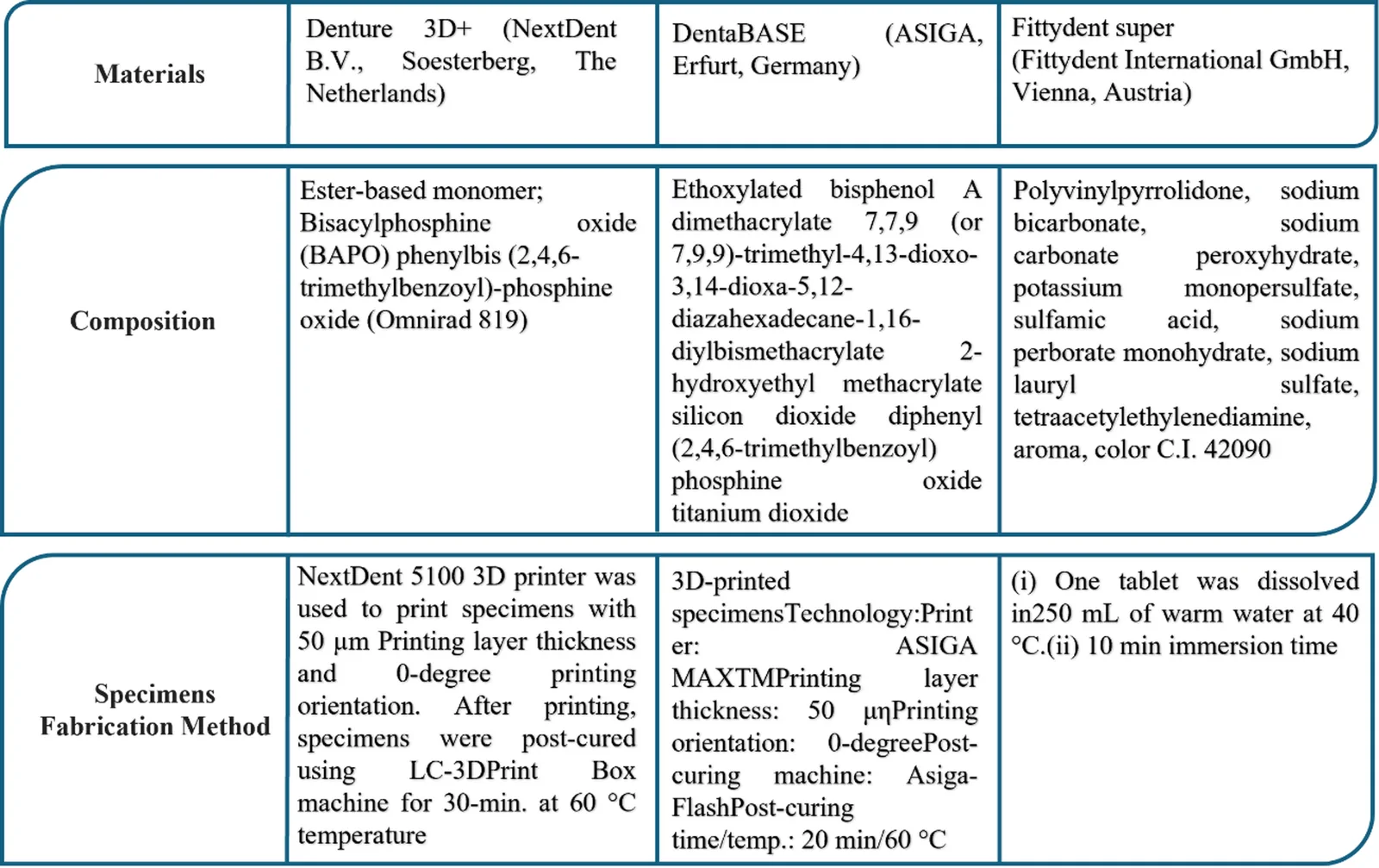
Materials used in the study and fabrication methods

Two 3D printed resins were used for a total of 200 samples preparations. DentaBase (Lot #5000533; Asiga, Erfurt, Germany) and Denture 3D+ (Lot #WU154N01; NextDent B.V., Soesterberg, The Netherlands).and printed with 0-dgree orientations and 50 μm layer thickness followed by post curing conditions. Materials used in present study and equipment as well as printing parameters and post-curing condition were detailed in (Fig. 1). For each resin, the printed samples were randomly subdivided into two groups on the basis of the duration of disinfection: 90 days or 180 days. One control group (no disinfection application) and four experimental groups (*n* = 20) were established according to the disinfection technique and denture material used, with a total of 10 groups as follows: control group; effervescent denture cleanser tablets fittydent (EDC) group; ultrasonic (US) group; ultraviolet (UV light group; and microwave (MW) group.

### Sample design and preparation

**Fig. 2:**
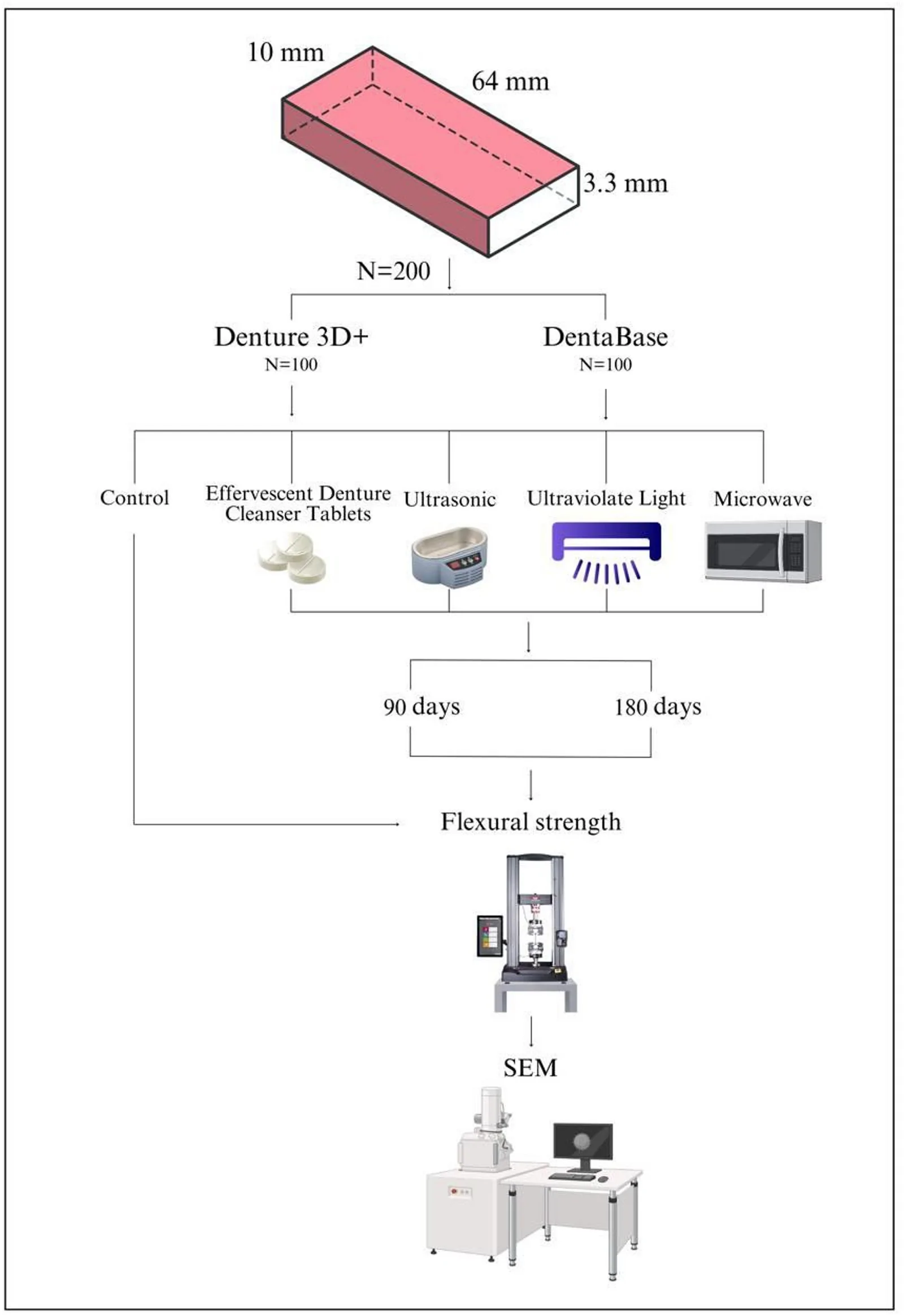
Illustration of study methodology *Disinfection protocols*

To calculate the sample size, an online calculator for power analysis was used. To compute the sample size, mean and standard deviation was obtained from a previously published article. The power was set as 80% and level of significance was set as 0.05. Hence, 10 samples per group are needed, resulting in a total of 200 samples, according to the resin type and disinfectant protocol. The samples were designed to have the required dimensions (bar-shaped samples, 64 × 10 × 3.3 mm) according to previous study via open-source CAD software (Autodesk Fusion 360, Autodesk Inc., USA) (Fig. 2). This process is formed as an Standard Tessellation Language (STL) file. The STL file was imported into each printer and printed according to the manufacturer’s recommendations, followed by the polishing using silicon carbide abrasive papers with different grits (500, 800, 1200) that mounted on an automated polishing machine (Metaserve 250 grinder-polisher; Buehler, Lake Bluff, IL, USA) C.

#### Chemical disinfection

The samples in the chemical disinfection group were immersed in 250 mL of ultrapure water containing one dissolved tablet of a commercial denture cleaner (Disinfectant effervescent tablet; Fittydent International, GmbH, Wien, Austria) lot#556,062,027 for 10 min at room temperature pH = 9, representing a single day of disinfection. The procedure was repeated to simulate 90 and 180 days of daily use, with samples thoroughly rinsed between each cycle and a fresh tablet used for each immersion [20].

#### Ultrasonic disinfection (US)

The samples in the ultrasonic cleansing group were treated via an ultrasonic cleaner (Blazer Model 3800 (A) 88–90 Allen Blvd, Farmingdale, NY 11735) operating at a power output of 35 W and 42 kHz for 5 min, which represented one day of disinfection. The initial water temperature was 21 °C. Temperature measurements were recorded before and after each cycle-with a final temperature of 54 C-to ensure that the water did not reach the glass transition temperature of the material.

#### UV light disinfection

The samples in the UV light group were disinfected via germicidal UV tubes (EGK BL T8 15, UV-A LAMP 15 W, Sachsenstr, Germany) 450 mm wavelength placed inside a box lined with aluminum on all the internal surfaces to increase light reflection and exposure. Each sample was exposed to ultraviolet light for 20 min on one side, then inverted and irradiated for an additional 20 min on the opposite side at a distance of 50 cm [21, 22].

#### Microwave disinfection

The samples in the microwave disinfection group were immersed in 250 mL of ultrapure water and placed in a 600 mL glass beaker. The beaker was positioned on the rotating plate of a microwave oven (Toshiba, JLTE-PH2-AA1, Dubai, UAE) and irradiated at a power setting of 700 W for 3 min, which represented one day of disinfection. The water temperature was measured before 21 C and after each cycle to monitor thermal changes with a final temperature of 65 C. Fresh distilled water was used for each cycle to ensure consistency in temperature.

#### Testing procedures

**Fig. 3:**
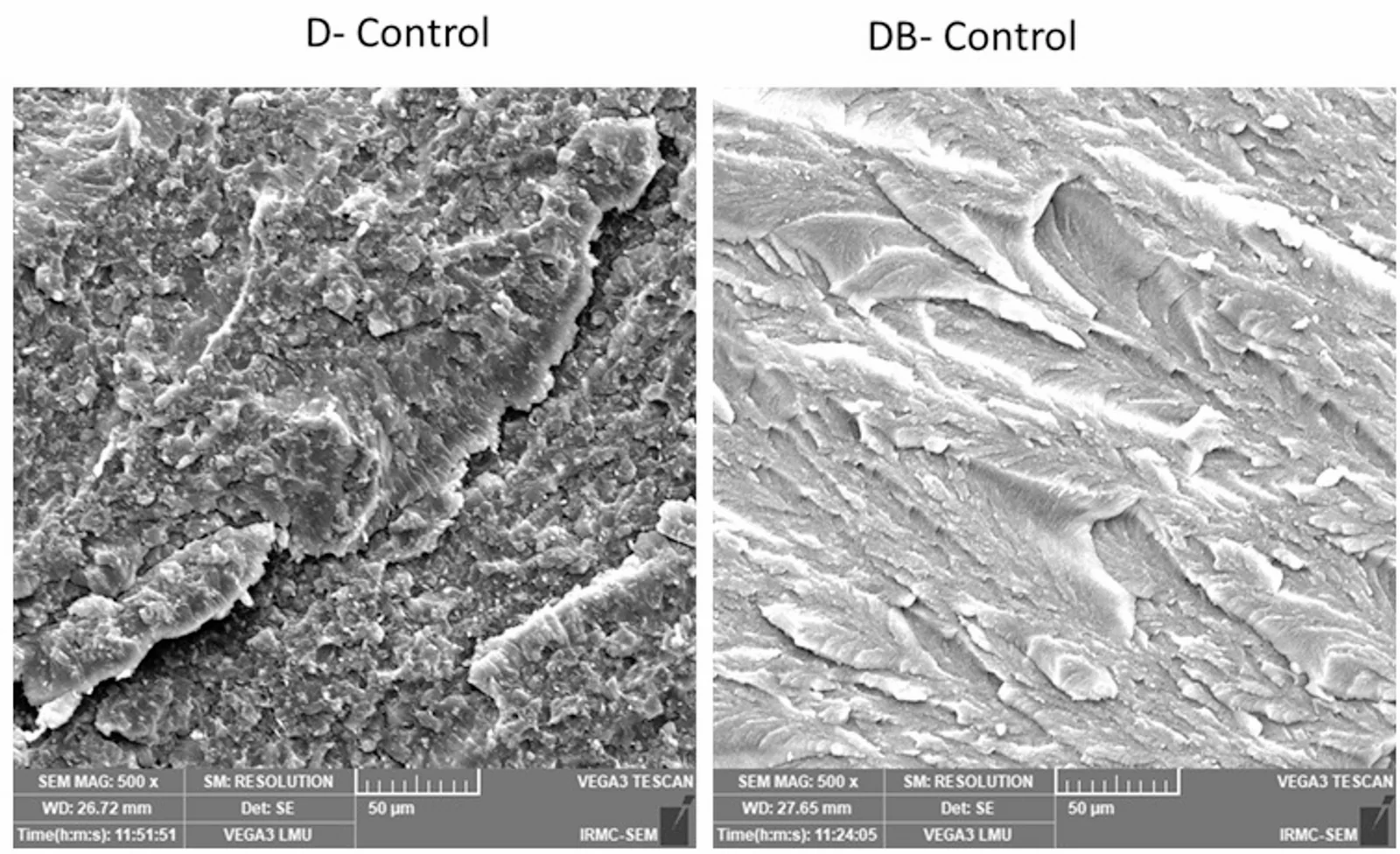
The SEM images of control group for Denture 3D+(D) and DentaBASE (DB) under x500

The 3-point bending test was conducted universal testing machine (Instron, Instron Corp. Norwood, MA, USA)) following previously documented protocols and ISO guidelines [22]. Each sample was placed on two vertical supports, 50 mm apart after being removed from the water. Until fracture occurred, the force was applied at the center of the sample at a crosshead speed of 5 mm/min. To determine the flexural strength, the fracture force (Newton) was measured [22]. After the fracture test, the fracture side was gold sputtered for analysis under a scanning electron microscope (FEI, Inspect S50, Czech Republic). The scanned areas were captured at different magnifications (x500–501) to illustrate the fracture behavior via various disinfection methods (Fig. 3).

### Statistical analysis

Statistical Package for Social Sciences SPSS, Version 23(IBM Corp.,New York, NY, USA). was used for analysis. The means and standard deviations were calculated for the descriptive analysis of the data. The Shapiro‒Wilk test was used to determine if the data were normally distributed, and insignificant results from the test provided that the data was normally distributed. For inferential analysis, parametric tests were employed using separate samples. A test compared the various meanings according to the time intervals. Additionally, and Three-way ANOVA was used to study the individual as well as combined effects on the factors on the flexural strength. and all *p* values less than 0.05 were considered statistically significant.

## Results

Table 1 shows a three-way ANOVA revealed significant main effects of method, time, and material individually (*p* < 0.001). A significant method × time interaction (*p* < 0.001) and a significant time × material interaction (*p* = 0.017). In contrast, the method × material interaction was not significant (*p* = 0.331), also no significant difference was observed with the combined effects of the three factors (*p* = 0.376).

**Table 1:** Three-way ANOVA for the individual and interaction effects among the factors

Source	Type III Sum of Squares	df	Mean Square	F	*p*
Method	805.7	3	268.5	40.3	< 0.001*
Time	829.7	2	414.8	62.3	< 0.001*
Material	935.36	1	935.3	140.5	< 0.001*
method * time	456.2	6	76.0	11.4	< 0.001*
method * material	22.9	3	7.6	1.1	0.331
time * material	55.5	2	27.7	4.2	0.017*
method * time * material	43.1	6	7.2	1.1	0.376
Total	1247548.6	240			

*statistically significant at 0.05 level of significance

Table 2 shows the mean values, standard deviations, and significance of the flexural strength for Denture 3D+. Statistical analysis revealed that in the EDCdisinfection group, the flexural strength of the Denture 3D+ denture base decreased significantly before and after disinfection at both the 90-day and 180-day intervals (*p* < 0.05), with the lowest flexural strength value at 180 days (65.1) compared with the US and UV values. On the other hand, US, UV, and MW disinfection groups were not significantly different from the control group at 90 days, with the highest flexural strength in the UV group (72.1%) (*p* < 0.013). However, at 180-day intervals, the US and MW groups demonstrated a significant decrease in flexural strength (*p* < 0.05).

**Table 2:** The flexural strength of Denture 3D+ with different disinfection methods

Interval	No (Control)	Disinfection method				*p*
		EDC	US	UV	MW	
90-days	71.7(1.8)^a^	67.9(2.3)^a, b^	70.8(3.3)	72.1(3.9)^b^	69.6(2.2)	
180-days	71.7(1.8)^a, b,c^	65.1(1.0)^a, d,e^	68.9(1.9)^b, d,f, g^	73.3(1.6)^e, f,h^	65.4(2.2)^c, g,h^	
*p*	-	0.002*	0.131	0.398	0.001*	

*ESC* Effervescent denture cleanser tablets, *US* Ultrasonic, *UV* Ultraviolet, *MW* Microwave

*statistically significant at 0.05 level of significance

Same small letters in each row showed statistical significance between the pairs by using Tukey Post hoc test

For the DentaBase (Table 3) denture base material, the EDCand MWdisinfection groups presented a statistically significant reduction in flexural strength compared with the control group and the UV group at 90-day intervals (*p* < 0.001). At 180-day intervals, the EDC, US, and MW disinfection methods significantly decreased the flexural strength (*p* < 0.001). However, no statistically significant difference was observed between the UV disinfection group and the control group at either time point (*p* = 0.22).

**Table 3:** The flexural strength of DentaBASE with different disinfection methods

Interval	No (Control)	Disinfection method				*p*
		EDC	US	UV	MW	
90-days	77.0(3.0)^a, b^	70.9(3.4)^a, c^	74.2(2.9)	77.8(3.2)^c, d^	70.6(2.5)^b, d^	
180-days	77.0(3.0)^a, b,c^	68.3(2.2)^a, d,e^	73.2(3.1)^b, d,f^	76.1(3.0)^e, g^	68.2(1.4)^c, f,g^	
*p*	-	0.061	0.465	0.220	0.017*	

*ESC* Effervescent denture cleanser tablets, *US* Ultrasonic, *UV* Ultraviolet, *MW* Microwave

*statistically significant at 0.05 level of significance

Same small letters in each row showed statistical significance between the pairs by using Tukey Post hoc test

**Fig. 4:**
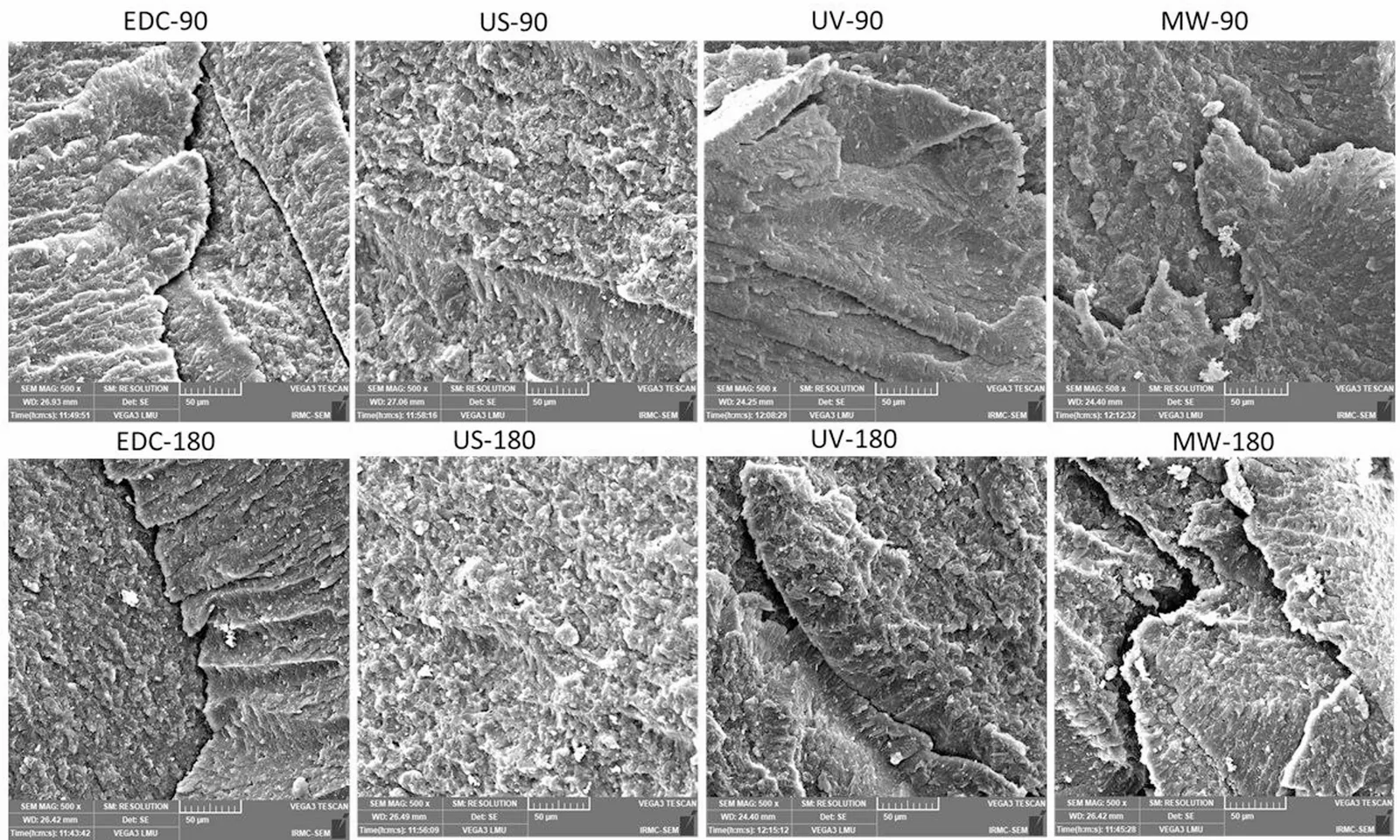
SEM images of disinfection groups for Denture 3D+ under x500 (ESC) Effervescent denture cleanser tablets (US), Ultrasonic, (UV) Ultraviolet, (MW) Microwave

**Fig. 5:**
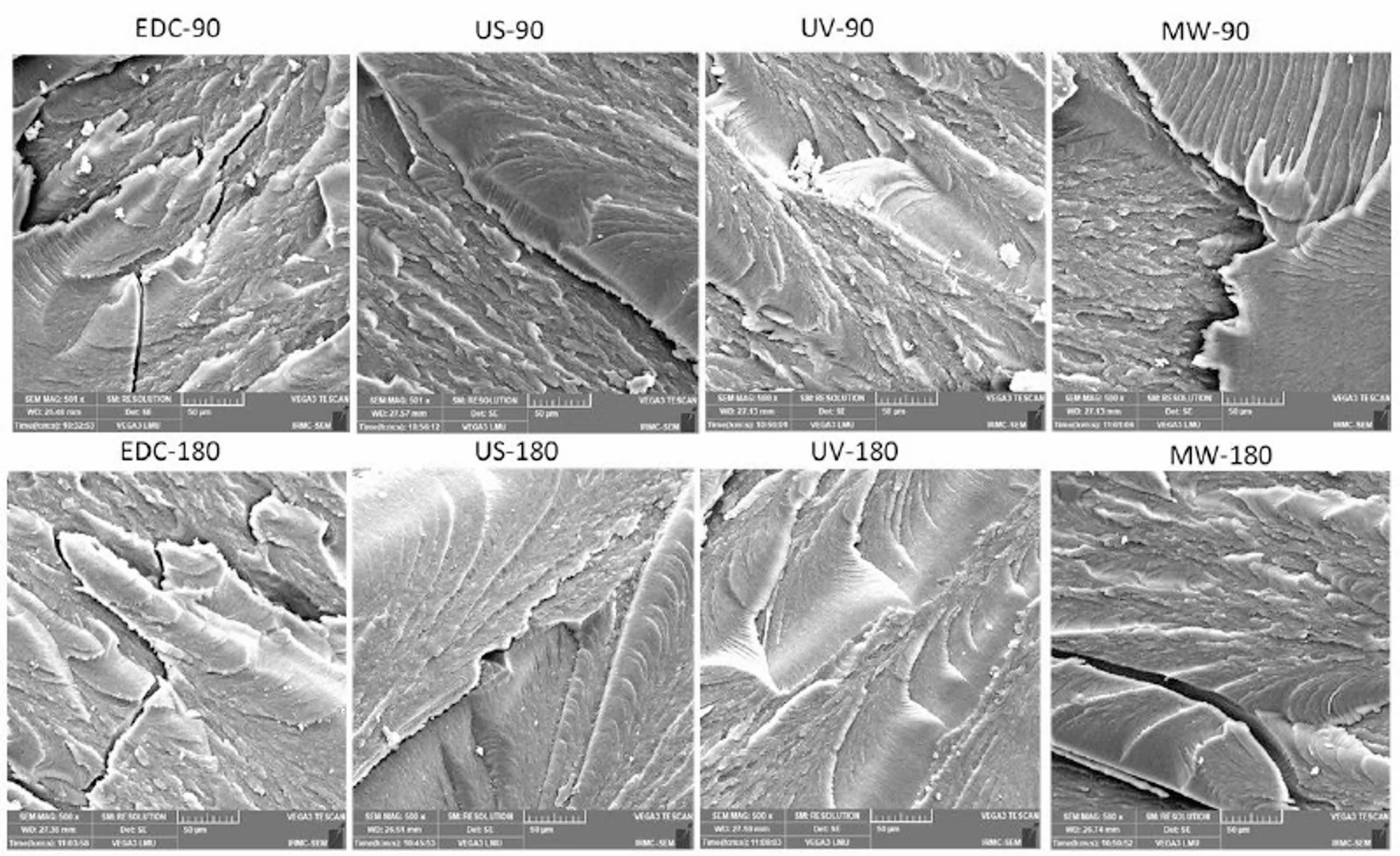
Show the SEM images of disinfection groups for DentaBase under x500 (ESC) Effervescent denture cleanser tablets (US), Ultrasonic, (UV) Ultraviolet, (MW) Microwave

Figure 3 shows representative SEM images of the control group (samples without disinfection). Both resins exhibited a ductile fracture mode due to the irregularity of the fracture side with the presence of sharp lamellae and grooves, which appeared in the DentaBase group compared with some faint lamellae in the Denture 3D+ group. Figures 4 and 5 illustrate the Denture 3D + and DentaBase groups obtained via various disinfection methods and time intervals. Both resins showed the same behavior in terms of SEM surface analysis. With EDC and MW, faint lamellae appeared, accompanied by cracks; some areas exhibited a mirror-like appearance. In the US, fewer lamellae and cracks disappear with a more irregular surface, representing an intermediate to ductile failure mode. On the other hand, UV light caused most of the fracture surface to have deep lamellae, and the flake-like appearance exhibited a ductile fracture mode.

## Discussion

Following various cleaning techniques, this study evaluated the flexural strength of base resins used in 3D-printed dentures. The null hypothesis was partially rejected because several disinfection techniques had a significant effect on the flexural strength of the 3D-printed polymers used to fabricate denture bases. A previous study demonstrated that the colony count of *C. albicans* might be successfully decreased by the use of chemical denture cleansers daily, highlighting their ease of use, especially for geriatric patients [23]. A study by Bae et al. [24] reported a 100% death rate of *C. albicans* when it was used at a dose of 1 tablet of Fittydent. Additionally, an in vitro study suggested that Fittydent, a regular chemical cleaning agent, could be used for long-term use as an alternative to denture cleaners on the basis of its effect on the physical properties of denture bases [25]. Significant increases in denture cleaning and patient satisfaction were observed among geriatric patients who used a portable vibrating ultrasonic bath with a chemical cleaner compared with those who used a chemical cleaner alone [26].

The potential of UV light devices as proper instruments for improving denture hygiene in the future is highlighted by their effectiveness in lowering microbial contamination on denture surfaces [27]. UV germicidal irradiation, which operates within the 200–320 nm wavelength range, is commonly used to disinfect air and surfaces [28]. In the study conducted by Pugazhendhi et al. [28], an UV disinfection unit operating under dry conditions was used to expose the denture samples to UV light at a wavelength of 254 nm for five minutes. A significant reduction in microbial contamination was detected, as indicated by the post disinfection colony counts.

Microwave disinfection is recognized as an efficient and time-saving method for denture disinfection in clinical dentistry [29]. At low energy levels, the thermal effect relies on water or ionic molecules exerting a bactericidal effect [30]. Choudhry et al. [7] demonstrated that microwave-treated samples significantly reduced the CFU count of *C. albicans* after just 3 min of exposure. Research has demonstrated that microwave disinfection alters the structure and permeability of fungal cells, thereby disrupting their metabolism and ultimately leading to cell death [31, 32]. These findings align with those of Al-Saadi et al. [33].

Water sorption on acrylic resins can significantly alter their physical characteristics and cause internal stresses, resulting in dimensional changes that compromise the long-term performance of dentures [34]. Berli et al. [35] reported higher water sorption values in 3D-printed resins. Minimizing water sorption and solubility is crucial in reducing the risk of fractures and cracks, thereby increasing denture stability [36]. Increased water sorption contributes to the development of internal stress and crack formation over time, ultimately affecting mechanical properties, particularly color stability [37]. These findings are compatible with those of Perea-Lowery [38]. The presence of crosslinking agents, plasticizers, initiators, and soluble materials can increase water sorption [39]. Chemical composition differences between heat-polymerized and 3D-printed resins also influence this property [38]. Gad et al. [40] reported that, compared with heat-polymerized resins, all 3D-printed resin groups presented greater water sorption and solubility, as well as lower translucency.

Additionally, in 3D printing, water can penetrate between layers and diffuse into the polymer matrix of the resin, filling interpolymeric spaces and pushing polymer chains apart [41, 42]. These observations support our findings, which indicate that UV light disinfection was performed under dry conditions, unlike other disinfection methods, which are typically conducted under wet conditions. This difference in experimental environments may explain why the flexural strength values of the UV-infected samples were similar to those of the control samples.

Although 3D-printed denture bases exhibit lower flexural strength than those fabricated via milled CAD-CAM technology do, they still meet the minimum ISO 20795-1 standard for flexural strength (≥ 65 MPa) [43]. The reduced mechanical performance of 3D-printed resins is attributed primarily to a lower degree of polymerization and the presence of residual monomers [44]. Additionally, 3D-printed acrylic resins exhibit a lower double-bond conversion rate, which negatively impacts their mechanical properties [45]. The inherent layer-by-layer fabrication process makes these materials more susceptible to void formation and incomplete particle fusion, especially during postcuring [46, 47]. With disinfection procedures, 3D-printed resins may be more negatively affected and display lower clinical performance in terms of strength. On the basis of these material differences, the present study aims to investigate the effects of various disinfection methods on the flexural strength of 3D-printed denture bases and to determine whether these methods could reduce the strength below the ISO threshold.

In this study, the flexural strength was significantly reduced when a denture cleanser was used to disinfect the 3D-printed denture base in Denture 3D + and DentaBase. Shah et al. [48] reported that denture cleaners decreased the flexural strength of polymethyl methacrylate (PMMA) denture base resin compared with water immersion. Other studies have reported similar findings and shown a reduction in the flexural strength of PMMA resins when exposed to peroxides and hypochlorite [48, 49]. The reduction in the flexural strength might be attributed to the longer duration of the current study, i.e., simulated 180-day usage.

This study evaluated the effects of ultrasonic disinfection on the flexural strength of 3D-printed denture base resins. The samples were subjected to ultrasonic disinfection for 90–180 min. After 90 days, no statistically significant difference in flexural strength was observed between the disinfected and control groups. However, a significant reduction in strength was noted after 180 days. This decline may be attributed to water sorption, as 3D-printed resins exhibit greater water sorption and solubility than conventional resins do. Additionally, the decrease in strength may be related to the consumption of all residual monomer through repeated disinfection cycles. The effect of ultrasonic disinfection on the flexural strength of denture base materials is unknown. Further comprehensive investigations are recommended to validate these findings.

In the current study, UV light disinfection did not result in a statistically significant difference in flexural strength for either DentaBase or Denture 3D+ denture base materials compared with the control groups. Bulut et al. [50] obtained similar findings by evaluating eight different disinfection methods, including microwave and UV light. Their results indicated that UV disinfection had the least detrimental effect on PMMA, particularly in terms of flexural strength. Among the tested methods, Bulut et al. reported that, among other disinfection methods, UV sanitization had the least adverse impact. This result may be attributed to the water sorption effect previously discussed. According to the SEM image, UV light results in the most fractured surface, characterized by deep lamellae and a flake-like appearance, indicating a ductile fracture mode, which supports our findings regarding the flexural strength of UV light.

The present study revealed that microwave disinfection significantly reduces the flexural strength of 3D-printed denture bases. This finding may be due to the consumption of all residual monomers in the previous cycles [51]. In a study by Hamouda et al. [52], the findings showed that the load required to fracture the samples decreased because of the microwave oven sterilization approach. Nevertheless, Consani et al. [53] reported no statistically significant difference (*p* > 0.05) in flexural strength between materials that were not disinfected and those that were disinfected. This difference in results may be attributed to variations in methodology and microwave disinfection settings. In the study by Consani et al., [53] a microwave with a power output of 650 W was used, with the samples immersed in 150 mL of distilled water. Additionally, the samples were subjected to five simulated disinfection cycles—one per day over five consecutive days—which differs from the protocol used in the present study. Ezzeldin et al. [51] concluded that microwave disinfection is a safe technique for 120 cycles that does not affect the flexural strength of CAD/CAM milled resin. However, an adverse effect on the flexural strength of 3D-printed resin was observed.

According to our findings, Denture 3D + and AS denture base resins exhibit similar behavior in terms of flexural strength following long-term exposure to various disinfection methods. These results indicate that the choice of disinfection method has a greater impact than the type of denture base material itself. Our data revealed that the combined effects of all three factors, time, disinfection method, and material, did not have a statistically significant association. However, the interactions between disinfection methods and materials were found to be significant. These findings support the conclusion that, after long-term disinfection (i.e., 180 cycles), the disinfection method, rather than the resin type, is the critical factor influencing flexural strength. Nonetheless, differences between the two types of resin can be observed during shorter-term disinfection cycles, such as those lasting 90 days.

When the disinfection methods were compared, no significant difference was observed between the microwave and chemical disinfection methods for the Denture 3D+ denture base. Similarly, ultrasonic disinfection did not significantly differ from chemical disinfection at 180-day intervals. UV light disinfection consistently resulted in higher FS values than those of the baseline and chemical disinfection groups.

For the DentaBase denture base, similar trends were observed. There was no significant difference between the chemical and microwave disinfection methods. Compared with chemical treatment, a reduction in FS with ultrasonic disinfection was evident only at 180 days. On the other hand, UV disinfection resulted in flexural strength values comparable to those of the control at both time points. These findings suggest that, regardless of the denture base material, UV disinfection does not negatively impact flexural strength. While other disinfection methods caused a reduction, all tested groups remained above the ISO 20795-1 minimum flexural strength requirement (≥ 65 MPa).

The strength of the present study lies in its ability to examine several denture base resins after different disinfection methods and intervals are applied. This comparative study can help clinicians choose the most appropriate disinfection methods for denture sterilization. However, its limitations as an in-vitro study stem from the absence of a complete intraoral environment, including intraoral flora, fluctuations in saliva pH, and occlusal forces. Additionally, only a 0-degree orientation was examined because printing orientations for 3D-printed resins alter the material properties, and the tested samples did not mirror the denture arrangements. However, further studies are needed to test the effects on *C. albicans*. Moreover, further investigations of different denture base resins are recommended.

This study has several limitations that should be considered when interpreting the results. All experiments were conducted under in vitro conditions, which may not completely reflect the material’s clinical behaviour. Moreover, only the flexural strength was evaluated, while other important properties such as water sorption and solubility, degree of conversion measured by fourier transform infrared spectroscopy, and glass transition temperature were not assessed. Future investigations should include these analyses to provide a more comprehensive understanding of the mechanical and physicochemical characteristics of 3D-printed denture base resins.

## Conclusion

Within the limitations of this study, a significant reduction in flexural strength was observed in both materials when subjected to chemical, microwave, and ultrasonic disinfection methods. However, all values remained within the acceptable range defined by ISO recommendation. UV disinfection consistently produced the highest flexural strength, with values comparable to those of the control group, making it the most favourable method for this application. Therefore, UV light can be recommended as an effective disinfection method for 3D-printed denture base resins. In addition, both resin types demonstrated similar flexural strength behaviour as the number of disinfection cycles increased.

## Data Availability

All data generated or analyzed during this study are available from the corresponding author on request.

## Abbreviations

DB: DentaBase
D: Denture 3D+
EDC: Effervescent Denture cleanser tablet
MW: Microwave
UV: Ultraviolet Light
US: Ultrasonic
FS: Flexural strength
